# Estimating direct and spill-over impacts of political elections on COVID-19 transmission using synthetic control methods

**DOI:** 10.1371/journal.pcbi.1008959

**Published:** 2021-05-27

**Authors:** Jue Tao Lim, Kenwin Maung, Sok Teng Tan, Suan Ee Ong, Jane Mingjie Lim, Joel Ruihan Koo, Haoyang Sun, Minah Park, Ken Wei Tan, Joanne Yoong, Alex R. Cook, Borame Sue Lee Dickens

**Affiliations:** 1 Saw Swee Hock School of Public Health, National University of Singapore and National University Health System, Singapore, Singapore; 2 Department of Economics, University of Rochester, Rochester, New York, United States of America; 3 Research for Impact, Singapore, Singapore; 4 The Galen Centre for Health and Social Policy, Kuala Lumpur, Malaysia; 5 Center for Economic and Social Research, University of Southern California, California, United States of America; University of California Irvine, UNITED STATES

## Abstract

Mass gathering events have been identified as high-risk environments for community transmission of coronavirus disease 2019 (COVID-19). Empirical estimates of their direct and spill-over effects however remain challenging to identify. In this study, we propose the use of a novel synthetic control framework to obtain causal estimates for direct and spill-over impacts of these events. The Sabah state elections in Malaysia were used as an example for our proposed methodology and we investigate the event’s spatial and temporal impacts on COVID-19 transmission. Results indicate an estimated (i) 70.0% of COVID-19 case counts within Sabah post-state election were attributable to the election’s direct effect; (ii) 64.4% of COVID-19 cases in the rest of Malaysia post-state election were attributable to the election’s spill-over effects. Sensitivity analysis was further conducted by examining epidemiological pre-trends, surveillance efforts, varying synthetic control matching characteristics and spill-over specifications. We demonstrate that our estimates are not due to pre-existing epidemiological trends, surveillance efforts, and/or preventive policies. These estimates highlight the potential of mass gatherings in one region to spill-over into an outbreak of national scale. Relaxations of mass gathering restrictions must therefore be carefully considered, even in the context of low community transmission and enforcement of safe distancing guidelines.

## Author summary

Mass gathering events have been identified as high-risk environments for community transmission of coronavirus disease 2019 (COVID-19). Empirical estimates of their direct and spill-over effects however remain limited. We frame the Sabah state election in Malaysia as a natural experiment to investigate the event’s spatial and temporal impacts on COVID-19 transmission. We estimate the direct and spill-over impact of the elections through a synthetic control methodology. Results indicate an estimated (i) 70.0% of COVID-19 case counts within Sabah post-state election were attributable to the election’s direct effect; (ii) 64.4% of COVID-19 cases in the rest of Malaysia post-state election were attributable to the election’s spill-over effects. We demonstrate that our estimates are not due to pre-existing epidemiological trends, surveillance efforts, and/or preventive policies. These estimates highlight the potential of mass gatherings in one region to spill-over into an outbreak of national scale. Relaxations of mass gathering restrictions must therefore be carefully considered, even in the context of low community transmission and enforcement of safe distancing guidelines.

## Introduction

As of 10 November 2020, there have been over 60 million reported cases and over a million fatalities due to coronavirus disease 2019 (COVID-19) worldwide. [1] In the absence of a vaccine, non-pharmaceutical interventions such as lockdown orders, limits to large gatherings, and travel restrictions have been employed to curb the onward spread of severe acute respiratory syndrome coronavirus 2 (SARS-CoV-2). [2] Although these measures have been largely effective in suppressing disease transmission, [3] their inevitable gradual relaxation has led to resurgences of COVID-19 across many regions, including North America, Europe, and Asia. [4–6] A key driver of this resurgence is large in-person gatherings, such as religious services and public rallies, where physical distancing between individuals is virtually unavoidable. Although these gatherings have been identified as high-risk environments for community transmission, [7] the direct and spill-over effects of these events on COVID-19 case counts have not been explicitly quantified and their broader implications are not known.

Malaysia has experienced an approximately four-month period of near zero incidence across all states due to the nationwide implementation of strict lockdown measures, broadly termed as the Movement Control Order (MCO). However, surges in COVID-19 case counts were reported in almost all major cities nationwide after a state election was held in Sabah, a large East Malaysian state in northern Borneo that is home to over 3.9 million people [8]. The Malaysian electoral system mandates that registered voters must cast their votes in-person at their registered residential districts with very few exceptions for mail-in ballots or early voting (see Table A in S1 Text for policy summary). Recent administrative data indicates that around 54.6% of registered individuals in Sabah (Sabahans) live outside of their registered residence within Sabah state, and 7.6% of Sabahans have migrated to other administrative regions. [9] High volumes of inter- and intra-state movement [10] to and within Sabah were therefore recorded during the campaign period leading up to the Sabah state election day on 26 September 2020. Airlines increased flight frequencies to ferry politicians and voters between Peninsular Malaysia and Sabah. [10] Politicians outside Sabah state were documented travelling into and within Sabah with campaign workers for physical rallies, with a total of 257 rallies or gatherings approved. [11] On the election day itself, over 1.1 million voters turned out to vote physically at designated polling stations (see Table A in S1 Text for summary). [12]

Post-election increases in COVID-19 case counts have been reported in multiple states with a record high of 2188 on 24^th^ November 2020, comprising of 1539 clusters in Selangor, 112 in Perak, 82 in Kuala Lumpur and 31 in Sabah. [13] It has therefore become urgent to understand the implications of these events on COVID-19 transmission, especially as countries around the world have tentatively begun to relax restrictions on large-scale gatherings despite not fully suppressing COVID-19 transmission in their respective communities. [14] In this study, we explore the Sabah election as a case study to quantify the direct impact of the election on COVID-19 case counts within Sabah, followed by their spill-over effects to other regions. Our proposed approach differs from traditional modelling and epidemiological frameworks as we employed synthetic control methods. Synthetic control methods integrate epidemiological and census data together with mobility data from flight volumes to identify treatment and spillover effects of large gatherings on COVID-19 transmission.

First, we used a large, detailed group of state and district-level socio-demographic and epidemiological characteristics to generate synthetic controls for each district within Sabah state. The optimal synthetic control was taken to be the one which most appropriately matched the pre-intervention period epidemic trajectory and its district characteristics. This allowed us to suitably ascertain a baseline level of COVID-19 transmission where physical election had not occurred in Sabah. Next, using daily domestic flight volume data and distance between districts/states, we constructed geographically resolved spill-over matrices to identify the indirect impacts of the Sabah state election on COVID-19 transmission in all other locales across Malaysia.

## Results

### Epidemic trends before and after the Sabah election

**Fig 1:**
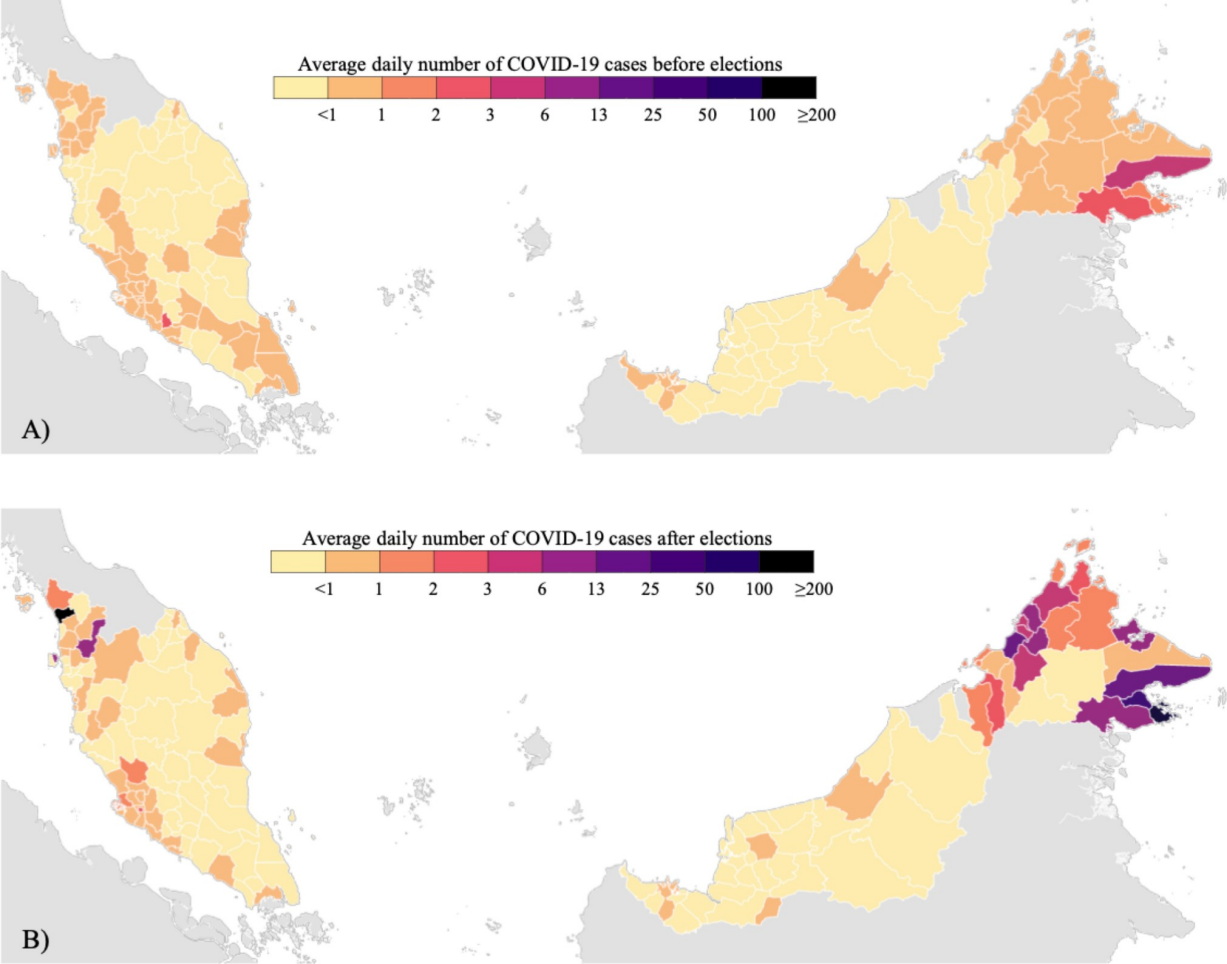
Panel A) Average daily number of COVID-19 case counts in district before Sabah election, after 10 June 2020. Panel B) Average daily number of COVID-19 case counts in district after Sabah election, before 12 October 2020. The figure was created using the base layer from The Humanitarian Data Exchange [15].

Nationally, Malaysia’s average daily number of new confirmed cases was low prior to the Sabah election, at around 16 reported cases per day (Fig 1A, 95% Quantile: 0, 59) from 10 June 2020 to 26 September 2020. For the following 17 days after the election and before the re-instatement of the nationwide lockdown policies (See Table A in S1 Text for summary), the average daily number of new confirmed cases was approximately 190 per day (Fig 1B, 95% Quantile: 49, 376). In post-election Sabah, the average daily number of new confirmed cases was 154 per day (Fig 1B, 95% quantile: 28, 343), and outside Sabah state, the average daily number of new confirmed cases was 37 per day (Fig 1B, 95% quantile: 3, 91).

### The impacts of the Sabah election on COVID-19 case counts

Across localities in Malaysia, epidemic trajectories may differ substantially due to naturally varying but related geographic and socio-demographic characteristics. We used synthetic control approaches to generate comparable control groups for districts within Sabah state and outside Sabah state to estimate the direct effects of the state election and spill-over effects across all other regions. [16,17]

**Fig 2:**
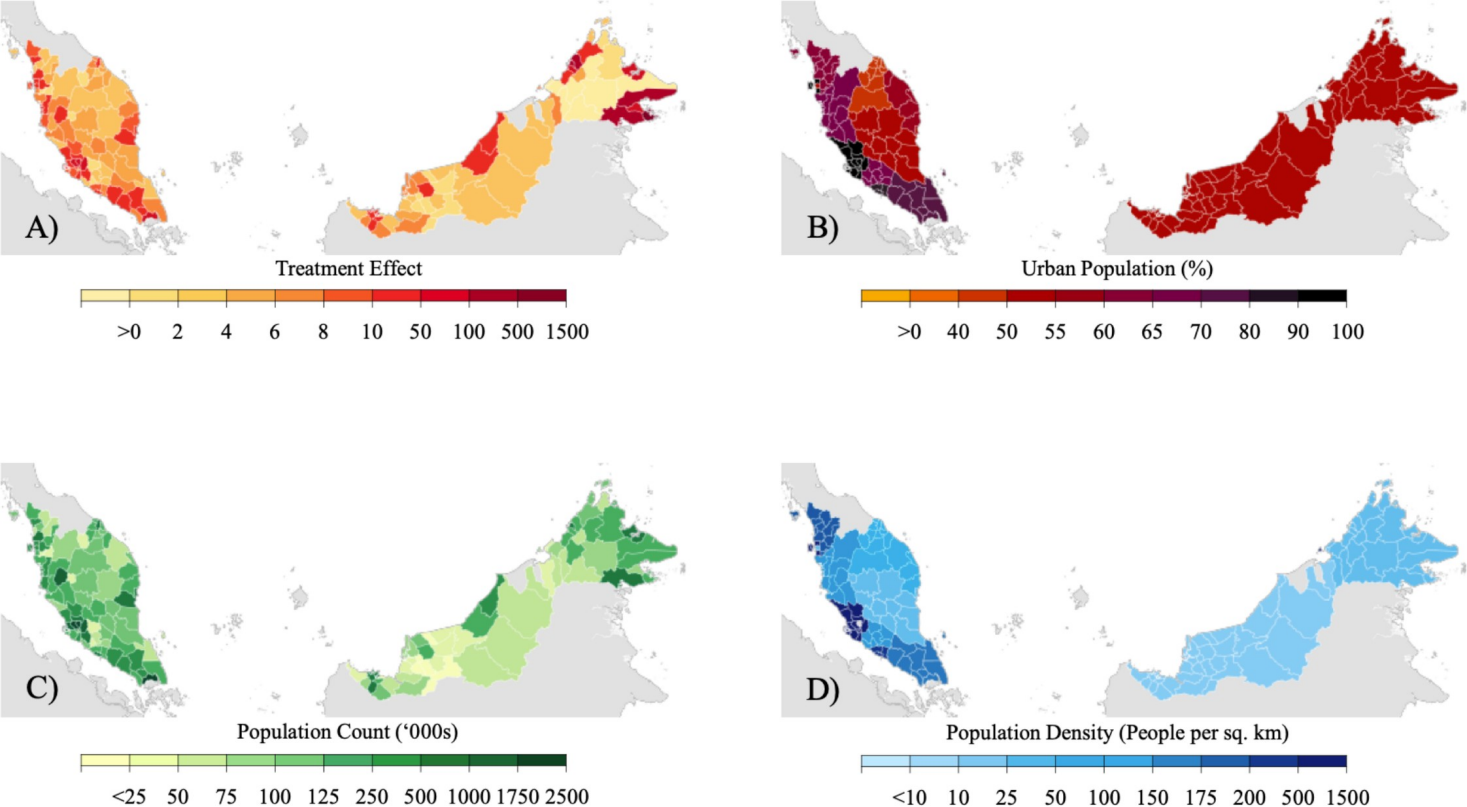
Panel A) Estimated treatment and spill-over effects of Sabah election on COVID-19 case counts in districts within Sabah State and districts outside Sabah State. Panel B) Urban population percentage within districts. Panel C) Population count within districts. Panel D) Population density within districts. The figure was created using the base layer from The Humanitarian Data Exchange [15].

When controlling for all state and district level characteristics, we estimate that 70.0% (Fig 2A, 95% CI: 34.4%, 105.6%) of COVID-19 cases after the Sabah election were attributable to the Sabah election, corresponding to a total of 2,979 COVID-19 cases (Fig 2A, 95% CI: 1817, 4142) for Sabah alone. The largest number of COVID-19 cases attributable to the election within Sabah were in Semporna, Tawau, and Lahad Datu districts, at 95.7% (Fig 2A, 95% CI: 61.7%, 129.7%), 79.5% (Fig 2A, 95% CI: 32.9%, 126.1%) and 96.7% (Fig 2A, 95% CI: 50.3%, 143.2%) respectively. Meanwhile, the smallest treatment impacts in COVID-19 cases due to the Sabah election within the state were negative and in Beaufort, Nabawan, and Kuala Penyu districts. However, districts with estimated effects in the negative direction accounted for less than 2% of total case counts pre- and post-election within the Sabah state. Among the 24 districts in Sabah where COVID-19 cases were reported, 70.0% were estimated to have increased case counts due to the election. All districts within Sabah with positive cumulative treatment impacts had 95% confidence intervals away from 0.

Similarly, post-election, we estimated that 64.4% (Fig 2A, 95% CI: 42.4%, 86.4%) of COVID-19 cases in non-Sabah districts of Malaysia were attributable to spill-over effects from the Sabah election, corresponding to 1,741 COVID-19 cases in total (Fig 2A, 95% CI: 1147, 2335). The largest number in COVID-19 cases due to the Sabah state election outside Sabah districts were in the urban Peninsular Malaysia districts of Petaling, Kuala Lumpur, and Johor Bahru, with a total of 124.7 (Fig 2A, 95% CI: 82.2, 167.2), 111.6 (Fig 2A, 95% CI: 73.5, 149.7) and 110 (Fig 2A, 95% CI: 72.5, 147.5) cases respectively. Among the 114 districts where COVID-19 case counts were reported outside Sabah state, all districts were estimated to have positive spill-over impacts with 95% confidence intervals for each cumulative spill-over impact away from 0.

### Impacts of matching characteristics and spill-over structures on treatment estimates

The choice of matching variables used may be subjective and can influence the construction of synthetic control weights for each region, thereby changing our treatment and spill-over estimates. As such, we conducted sensitivity analysis by exploring 10 000 different sets of matching variables to guard against potential sensitivities in adding or removing matching variables. Our results indicate that the Sabah state election led to positive increases in COVID-19 case counts in Sabah districts for 97.6% of the scenarios, with all 95% uncertainty interval (UI) for these scenarios not crossing the 0 bound (95% UI: 352, 4812). Similarly, spill-over effects were positive for non-Sabah districts for 98.7% of the scenarios. The 95% uncertainty interval for these scenarios also did not cross the 0 bound (95% UI: 107, 2188).

Although the magnitudes of spill-over effects are estimated based on data, the spill-over structures specified may be subjective. We explored three spill-over structures in our sensitivity analysis. When using all epidemiological, socio-demographic, and geographic characteristics, the spill-over effects on districts away from Sabah state were all positive in direction (Range: 1113, 2937). Hypothesis tests also indicated that the treatment and spill-over effects are significant and away from zero at the 5% level for each of these structures in 97.6% of cases, with these effects being positive in 90% of cases (See Fig H in S1 Text). This demonstrates that the impact of the election on other states in terms of point estimate uncertainty and direction on COVID-19 case counts are not sensitive to the form of spill-over structure imposed.

### Impacts of pre-existing epidemiological trends, surveillance and preventive policies on treatment estimates

Pre-existing epidemiological trends, surveillance, and preventive policies may skew treatment estimates. We created placebo events 4 to 8 weeks before the actual election occurred and estimated treatment and spill-over effects from these placebo events to ascertain the existence of pre-existing epidemiological trends. In all 5 cases, cumulative point estimates for the direct treatment effects on Sabah state for 3 weeks after these placebo events were small (Range: –65.2, 377.5). Similarly, cumulative point estimates for the spill-over effects away from Sabah state for 3 weeks after these placebo events were small (Range: 31.4, 90.4). Hypothesis tests for the presence of treatment and spill-over effects found that 91.7% and 100% of the time points, after all placebo events had occurred, had treatment and spill-over effects which were statistically indistinguishable from 0. This demonstrates that our estimates for the actual treatment and spill-over effects of the Sabah election were unlikely to be due to pre-existing trends.

We cut off estimates for the treatment and spill-over effects of Sabah state election at 12 October 2020 due to state-wide lockdown policies being enacted after that time point. Our review of national and state-level non-pharmaceutical intervention policies in Malaysia showed no enactment of strict social distancing policies between 25^th^ September 2020 to 12^th^ October 2020 outside Sabah state (See Table A in S1 Text).

**Fig 3:**
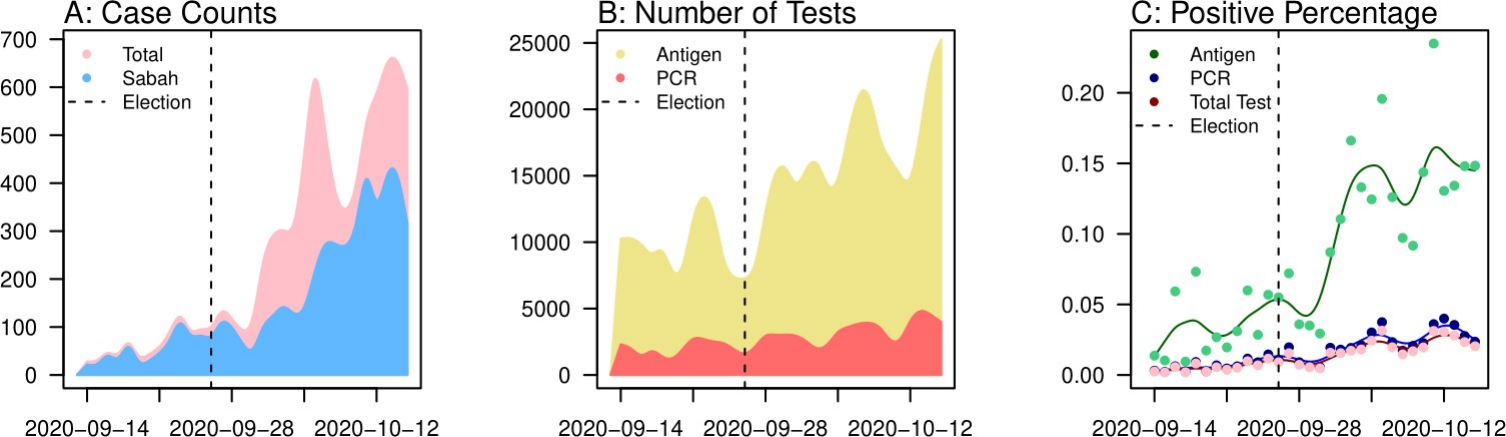
(A) Reported case counts from 9^th^ September 2020 to 10^th^ October 2020 for Sabah State and nationwide (B) Number of polymerase chain reaction (PCR) and antigen tests conducted from 9^th^ September 2020 to 10^th^ October 2020 nationwide (C) Percentage tested positive for polymerase chain reaction (PCR) and antigen tests conducted from 9^th^ September 2020 to 10^th^ October 2020 nationwide.

Although heightened surveillance from test-and-trace policies may have inflated treatment and spill-over effect estimates, we ascertained that this is not the case. First, while there was an apparent increase in the number of tests conducted nationwide post-Sabah election, with an average of 19,458 tests per day (Fig 3, Range: 7,619, 24,952), as compared to the pre-election figure of 11,909 tests per day (Fig 3, Range: 8,667, 15,978), the test positive rate has also increased from 0.6% to 2.2% for polymerase chain reaction (PCR) tests and 3.4% to 11.5% for antigen tests. This points to a proportionally larger increase in the number of cases compared to the increase in number of tests, indicating that case counts have increased beyond what was expected from increased testing rates. This would otherwise hold if test positive rates were constant before and after the Sabah election. Second, population-wide proactive testing campaigns were not documented to have occurred during this time. As such, the post-election increase in case counts are unlikely to be due to changes in reporting rate. Lastly, Malaysia conducts primarily manual contact tracing as a first-line preventive strategy when the number of COVID-19 cases is low. [18] This was likely to have reached capacity limits post-Sabah election, which rules out the possibility of our treatment effect estimate being due to active case-seeking.

## Discussion

Using the Sabah state election as a case study, our analysis demonstrates both the direct risk of COVID-19 transmission from mass gatherings and the potential for these mass gatherings to spill-over to other regions, resulting in an outbreak of national scale. We found that the estimated direct impact of the Sabah state election on Sabah state’s COVID-19 transmission and estimated spill-over impacts to other regions are robust to the types of matching variable used to generate the baseline transmission level. These estimates are also robust to the types of metrics used to infer the spill-overs effects to other regions. We further showed that these estimates are not due to pre-existing epidemiological trends or heightened surveillance measures implemented post-election.

Although the risks of holding large gatherings during the COVID-19 have been highlighted in scientific literature, [7,19] limited information exists on the quantified risk of allowing such gatherings. The risk pre-election of an outbreak was relatively low with Sabah reporting low, but non-zero, transmission of COVID-19 in the community [8] and high voter compliance to mask-wearing practices, physical distancing, and conducting temperature checks. [20] During the election period, test positives were not allowed to vote whilst symptomatic and quarantined individuals were able to vote in designated balloting areas, [21] of which the latter minimised the risk of potential infections coming into contact with susceptible individuals. Factors which conversely increased transmission risk included the permitting of physical rallies of unlimited attendees in open spaces and up to 250 individuals in enclosed spaces [22] Additionally, campaigning politicians, their entourages, and rally attendees were reported to have had poor adherence to safe distancing and mask-wearing guidelines at events. [23,24] Although policies were rapidly changed, limiting attendee numbers to 30 for political talks, [25] the high transmissibility of COVID-19 and cumulated increased risk of infection spread may have been sufficient to lead to increased post-election COVID-19 case counts within the state.

Our results also show a relationship between the substantial increase in case counts in other regions of Malaysia with the Sabah state election. Lockdown policies were relaxed in early May 2020 and interstate travel in Malaysia was allowed from 10 June, except for regions deemed at risk of COVID-19 transmission. [26] In Sabah state, interstate travel was allowed during the election period, with in-person voting mandated within each individual’s registered district: this led to an influx of around 250,000 Sabahan voters returning from other parts of Malaysia. Additionally, politicians and campaign workers flew in from Peninsular Malaysia to support their counterparts. [27] However, post- state election, it was not mandatory for voters coming to Sabah from other parts of Malaysia to undergo 14-day quarantine upon return to their states of residence; only those returning from Sabah’s red zone areas or those displaying symptoms were encouraged to be swab-tested. [28] It is therefore plausible that some of these interstate travellers contracted COVID-19 during the election period due to increased intensity of mixing, and that these individuals may have played roles in seeding new outbreaks in other states upon their returns from Sabah. In particular, spill-over impact estimates from the Sabah election were largest in the Peninsular Malaysia states of Selangor, Kuala Lumpur, and Johor. These states, which have large urban centres and are common destinations for both inter- and intrastate emigrants, [29] also host the highest percentages of emigrant Sabahans. [9] This demonstrates the increased risk of COVID-19 spill-overs to other regions due to the Sabah state election, given higher volumes in movement. [9]

Notably, context-specific COVID-19 control measures, like proactive testing, tracing, and quarantining, were absent for travellers into and within Sabah during and after the election, despite records of increasing community cases within Sabah and the known risks associated with large gatherings. [30] This study thus demonstrates the need for strict social distancing measures with mass gathering events, which is particularly imperative as countries start to relax social distancing measures and policies on large gatherings and internal movement. [14] Available evidence on nationwide transitions in COVID-19 recovery prior to immunisation recommends a phased approach informed by context-specific data, prioritising community trust and resilience through decisive policies and clear communication. [31] Further, as alternatives to large-scale lockdown measures, multi-pronged strategies presented in previous literature include continued incorporation of COVID-19 testing in the community, rapid contact tracing, and localised quarantine. [32]

This study is subject to several limitations. First, only flight data from 2010 to 2017 was available to construct the spill-over matrices in our analysis, due to data availability issues. However, we show that our spill-over effect estimates were robust to different parameterizations of the spill-over matrices. The magnitudes of spill-over were also calibrated primarily on observed case counts across locales, rather than structure. Second, difference in reporting rates between districts may skew our treatment estimates. While explicit estimates for number of tests are not available across regions, our sensitivity analysis showed that the increase in number of tests did not outpace the growth in case counts. We also controlled for healthcare capacity in constructing our synthetic controls, to further adjust for reporting rates between regions. While death rates from COVID-19 are available at national level, our synthetic control framework requires listings over time for both treated and un-treated regions. When death rates across districts become available, the same synthetic control framework should ideally be calibrated separately on death rates, to further alleviate issues regarding the reporting rate between districts and understand the additional burdens caused by the Sabah election.

Third, the time point of treatment which is the Sabah state election was defined as a single date based on when polling took place. According to the Malaysian Federal Constitution, an election’s campaign period is gazetted as the 14-day period leading to the actual polling day and poll counting starts immediately after polls close. [23,33] Poll counting at the Sabah state election was completed on election day itself. Hence, this date was chosen as it is the definite cut-off for any further election activities. Our placebo treatment sensitivity analysis showed that there is no evidence of COVID-19 transmission pre-trends before election occurred. Fourth, we were unable to account for the impact of Sabah’s stateless residents and irregular migrants, who comprise over a quarter of Sabah’s resident population, on Sabah’s COVID-19 case numbers and transmission dynamics. [34] Many individuals work in the informal sector, live in the state’s rural interior or on remote islands, typically do not have access to resources accorded to citizens (e.g. civil registration, healthcare, education, government aid), and risk detention or deportation if discovered. [35] These circumstances therefore make it challenging to test, contact trace, screen, or collect robust, reliable data on these populations. Also, since August 2020, prisons and detention centres have been identified as key sources of outbreaks across Malaysia. [36] Although we recognise their influence on COVID-19 case counts in both Sabah and Malaysia more broadly, data limitations—including a lack of public case listing—meant we were unable to fully control for their effects in our analysis.

Quantifying the risks of COVID-19 transmission from large gatherings is inherently difficult due to the large number of competing, dynamic factors that influence transmission over time. Using the synthetic control approach, we created appropriate control groups for each locale to provide a what-if scenario for each locale had the Sabah state election not occurred. Our study found that there are severe direct and indirect implications of mass gatherings, using the Sabah state election as an anchoring event. In summary, these estimates highlight the potential of mass gatherings in one region to spill-over into an outbreak of national scale.

Our findings and their importance fall in line with the Malaysian federal government’s recent actions to prevent the events post-Sabah from repeating themselves. On 18 November, the Malaysian King declared a state of emergency for Sabah’s Batu Sapi parliamentary constituency, postponing its by-election which had been slated for 5 December. Prime Minister Muhyiddin Yassin said that in advising the King on his decision, the Cabinet had considered the severity of Sabah’s pandemic situation post-state election and the potential of further negative COVID-19 case count, socioeconomic, and health systems impacts following a by-election. [37] However, challenges remain. At time of writing, the Malaysian Election Commission had declared that the Batu Sapi by-election and other pending by-elections (i.e., Bugaya in Sabah and Gerik in the Peninsular Malaysia state of Perak) would be conducted physically in January 2021, [38] as the current electoral system is unequipped to manage large-scale postal voting. [39] Although more stringent guidelines will be implemented, including suspension of all face-to-face campaigning activities including rallies, house visits, and walkabouts, the extent to which these guidelines will be relevant, appropriate, or enforceable come next year remains unclear given the ever-shifting nature of this pandemic. Moving forward, relaxations of mass gathering restrictions must therefore be carefully considered, even in the context of low community transmission and enforcement of safe distancing guidelines.

## Methods

### Data sources

- **COVID-19 case count:** COVID-19 case count data is collected at national, administrative region and district resolutions by the Ministry of Health, Malaysia daily on official government websites and social media pages. [1,13] Case counts are reported to occur in all 13 administrative regions and over 150 districts as of 10^th^ November 2020. Data at the district resolution is available from 1 March 2020 to 10^th^ November 2020. We used the period from 22 March 2020 to 12 October 2020 for our analysis due to lockdowns and/or travel restrictions before and after those time points respectively.
- **COVID-19 testing data:** Nationwide COVID-19 testing rates using polymerase chain reaction and antigen tests were reported by the Ministry of Health, Malaysia from 14 September 2020 to 15 October 2020. [40] These were used for sensitivity analysis on the level of surveillance before and after the election occurred.
- **Socio-demographic data:** The Department of Statistics Malaysia conducts population and housing census at the district level every 10 years. We obtained district-level characteristics on population size, ethnicity composition and the total number of households from the 2010 Malaysian census. [8] State level characteristics on healthcare capacity such as the number of healthcare professionals, number of hospitals and diagnostic centers were reported by the Ministry of Health Malaysia and are publicly available from 2015 to 2018. Lastly, the exact locations of primary care clinics were reported for 2019 in Malaysia and was aggregated to both the district and state level. [8] These characteristics were used for the generation of synthetic controls.
- **Geographic data:** Spatial characteristics which may influence the spread of COVID-19 were considered. Population density and count was obtained from Socioeconomic Data and Applications Center (SEDAC) at a 1km gridded resolution where the former was averaged at the district level and the latter summated. [41] Spatial accessibility data in terms of travel time to the nearest population centre of at least 50,000 inhabitants was taken from Weiss et al. at a 1km gridded resolution where the range and average accessibility for each district was obtained. [42]
- **Malaysia mobility data:** Passenger flight volumes for 2013 to 2017 were available on a monthly time scale from the Official Aviations Guide. [43] This data was used to parameterize connectivity and movement between regions in Malaysia.
- **Metrics for large-gathering policies:** We obtained COVID-19 policy responses by using a publicly available database of state and national level COVID-19 response policies, which includes restrictions on the size of gatherings and on internal movements within the nation. Data was collected from publicly available sources such as news articles and official press releases and briefings. These are identified via internet searches and all original source material is archived and the coding of policy responses can be checked and substantiated. [14]

### Synthetic controls for Sabah state and districts

We examined the causal impact of state election on case counts in Sabah and the spill-over effects on districts outside of Sabah using synthetic control methods. For notation, we set the total number of periods observed to be *T* and the treatment date to be *t*_0_<*T*. Then, the impact of state election at time *t*_0_+*k* for *k* = 0,…,*T*−*t*_0_ and all districts is given by

$$\alpha_{t_{0}+k}\equiv Y_{t_{0}+k}(1)-Y_{t_{0}+k}(0),$$

where *Y*_*t*_(·) is a vector of cumulative case counts per 100 individuals for every district in Malaysia in our sample at time *t* (total sample size of *J*). Note that *Y*_*t*_(·) is a function of the treatment history, where a value of 1 indicates a history in which state election occurred in Sabah on date *t*_0_ while a value of 0 refers to the counterfactual scenario where no election were held. Note that we only observe $Y_{t_{0}+k}(1)$ and not $Y_{t_{0}+k}(0)$. Hence, we estimated $Y_{t_{0}+k}(0)$ by synthetic control methods generalized to the case where spill-overs are permitted. [16,17] Specifically, for each district *i*, we approximated its case counts at time *t* using a weighted combination of counts from other districts with similar predictor values: ${\hat{Y}}_{i,t}=\sum_{j\neq i}^{J}w_{j}^{i}Y_{j,t}$. The weights $W_{i}={\left(w_{1}^{i},\ldots ,w_{i-1}^{i},w_{i+1}^{i},\ldots ,w_{J}^{i}\right)}^{⊤}$, which are non-negative and sum to 1, are chosen such that the pre-election (i.e. *t*<*t*_0_) values of predictors for district *i* is close to that of predictors from other districts. Formally, for a norm ‖·‖, we estimate *W*_*i*_ as

$$W_{i}^{*}=\arg\;\min\left\|X_{i}-X_{j\neq i}W_{i}\right\|,$$

where ***X***_*i*_ (*K*×1) and ***X***_*j*≠*i*_ (*K*×*J*−1) contain predictors including past (i.e. prior to *t*_0_) COVID-19 case counts and, socio-demographic and mobility data, for district *i* and other districts respectively. With the optimized weights, we can construct ${\hat{Y}}_{t_{0}+k}={\left({\hat{Y}}_{1,t_{0}+k},\ldots ,{\hat{Y}}_{{J,t}_{0}+k}\right)}^{⊤}$ as an estimate of $Y_{t_{0}+k}(0)$.

### Inferring direct treatment and spill-over effects

Our investigation differs from the conventional synthetic control setting which relies on the Stable Unit Treatment Value Assumption (SUTVA). This would have implied that election in Sabah affected only districts in Sabah and yields no spill-over effects. This is unlikely to hold in the data due to the presence of individuals who travel to and leave Sabah before and after the election respectively. [11,27,30] Estimation with synthetic controls under the SUTVA framework in the presence of spill-overs will result in biased estimates. Hence, we followed Cao and Dowd (2019) [17] to accommodate spill-over effects in estimating ${\hat{\alpha }}_{t_{0}+k}$.

We decomposed the total treatment effect from the preceding section as $\alpha_{t_{0}+k}=A\gamma_{t_{0}+k}$, where ***A*** is a matrix specifying the spill-over structure, and $\gamma_{t}={\left(\beta_{1,t},\ldots ,\beta_{j_{1},t},b_{t}\right)}^{⊤}$ is a parameter vector containing the direct impact of state election on districts in Sabah at time *t* (*β*_*s*,*t*_ for *s* = 1,…,*j*_1_ districts) and the spill-over effect on districts outside of Sabah (*j*_0_ districts) is controlled by the parameter *b*_*t*_. Without loss of generality, we have arranged the first *j*_1_ entries in $\alpha_{t_{0}+k}$ to correspond to districts in Sabah. Then, we defined the spill-over matrix ***A*** such that for a district *s* in Sabah, the effect of state election is given by $\beta_{s,t_{0}+k}$, while the spill-over effect for district s* outside of Sabah is given as the product of its geometric distance measure exp(−*d*_s*_) between the district and Sabah with the spill-over parameter $b_{t_{0}+k}$. Taken together, ***A*** is defined as

$$A=\left(\begin{array}{cc} I_{j_{1}} & 0\\ 0 & exp(-d_{1})\\ \vdots & \vdots \\ 0 & exp(-d_{j_{0}}) \end{array}\right),$$

where $I_{j_{1}}$ is the *j*_1_-dimensional identity matrix. This parameterization allows the spill-over effect to decay as the geometric distance increases.

The parameter vector $\gamma_{t_{0}+k}$ for *k* = 1,…,*T*−*t*_0_ can be recovered by minimizing the error between the counterfactual outcome $Y_{t_{0}+k}(0)$ and the estimated synthetic control ${\hat{Y}}_{t_{0}+k}$. Note that even though $Y_{t_{0}+k}(0)$ is unobserved, we can, by definition, replace it with $Y_{t_{0}+k}(1)-\alpha_{t_{0}+k}$, which yields the following optimization problem

$${\hat{\gamma }}_{t_{0}+k}=\arg\;\mathrm{mi}n_{g}\|\left(I-\hat{B})(Y_{t_{0}+k}(1)-Ag\right)\|={\left(A^{⊤}\hat{M}A\right)}^{-1}A^{⊤}\hat{M}Y_{t_{0}+k}(1),$$

where $\hat{M}={\left(I-\hat{B}\right)}^{⊤}(I-\hat{B}),\;\hat{B}={\left(\hat{W_{1}},\ldots ,\hat{W_{J}}\right)}^{⊤}$ and $\hat{W_{i}}$ is the *J*×1 vector of synthetic weights for district *i* where the *i*^th^ entry is set at 0.

The total impact of state election on districts in Sabah at time *t*_0_+*k* can then be computed as $\sum_{j=1}^{j_{1}}\beta_{j,t_{0}+k}$. The associated 95% confidence interval is obtained by inverting the joint test with the null hypothesis of $H_{0}:\sum_{j=1}^{j_{1}}\beta_{j,t_{0}+k}=0$. Likewise, the 95% confidence interval for the spill-over effects can be obtained by testing $H_{0}:b_{t_{0}+k}=0$. Interested readers may refer to Cao and Dowd (2019) for further details. [17]

### Sensitivity analysis: Matching characteristics

Our primary matching variable was cumulative daily COVID-19 case counts per 100 individuals from 10 June 2020 (end of lockdown) to 25 September 2020 (day before Sabah election). Reported case counts were population rescaled to correct for differences in size and infection potential between regions. [44] Additionally, we considered matching in each region, state and district specific covariates. These include population, population density, urbanicity, number of registered vehicles, number of medical practitioners, number of diagnostic centres, number of hospitals and number of primary care clinics. We assessed the impacts of including these additional matching criteria by randomly subsetting the matching covariates used, in addition to daily COVID-19 case counts within each region to construct synthetic controls. We created synthetic controls using the subsetted dataset before the election occurs and computed each region’s respective direct treatment and spill-over effects after the election occurs 10 000 times. We compared the treatment and spill-over effects from the fully controlled data set comprising all observations and the treatment and spill-over effects from the subsetted data set by the fully controlled data set’s effect estimates on the density plot of the subsetted data set effect estimates.

### Sensitivity analysis: Placebo events

We constructed placebo treatment events for 4 to 8 weeks before the actual election occur. These placebo events were used to determine whether the measured treatment effects on the Sabah election on COVID-19 case counts are attributable to pre-existing trends. The synthetic controls were constructed based on data before the placebo treatment event occurs, but after the lockdown on 22 March 2020 for all regions to remove the presence of any prior policy effect on COVID-19 case counts. We measured the treatment and spill-over effects of these placebo events following **M2** and **M3**, for a total of 15 days after the placebo event, corresponding to the duration where treatment and spill-over effects were measured from the actual Sabah election.

### Sensitivity analysis: Spill-over matrices

To construct the effect matrix ***A***, we need knowledge of the geometric distance measure exp(−*d*_*j*_) between the spill-over and the treated region. We explored three parameterizations of the geometric distance metric, namely: (1) flight distance between each region’s primary airport to Sabah’s primary airport (2) number of flight passengers between each region’s primary airport to Sabah’s primary airport in October 2012 (3) number of flight passengers between each region’s primary airport to Sabah’s primary airport from 2010 to 2017.

These measures provide a measure of connectivity between the spill-over and treated regions. We used October 2012 to capture past Sabah election season flight patterns and the overall number of flight passengers to capture overall flight volumes between each state. Each measure was normalized to a scale of 0 to 1 by subtracting each value by the maximum of the observed values and divided by the range of observed values before computing the geometric distance measure in ***A*** to avoid numerical overflow.

### Sensitivity analysis: Impacts of co-current contract tracing on treatment effects

Co-current interventions efforts were in place after the Sabah election occurred, which included manual contact trace and test efforts, which have led to a surge in the number of tests being conducted nationwide. [18] We computed the test positive rate by dividing the number of reported COVID-19 cases nationwide and separately in Sabah district by the number of tests being conducted in 14 September 2020 to 15 October 2020. This was to ascertain whether the rise in case counts was due to increased surveillance and testing or by the election.

## Supporting information

S1 Text Appendix containing additional details on results. The figures were created using the base layer from The Humanitarian Data Exchange. **Table A. Malaysia policy summary. Fig A. 95% Confidence intervals for treatment/spill-over effects at the district level. Fig B. Matching variables used for generation of synthetic controls (Healthcare capacity). Fig C. Matching variables used for generation of synthetic controls (Demographic/Geographic). Fig D. Sensitivity analysis on matching characteristics: magnitude of treatment/spill-over effects. Fig E. Sensitivity analysis on spill-over matrix, number of significant regions. Fig F. Sensitivity analysis on spill-over matrix, number of regions with positive treatment effects. Fig G. Sensitivity analysis on spill-over matrix, cumulative treatment effect. Fig H. Placebo test for treatment and spill-over effects prior to Sabah election.**
(DOCX)
