# Supplementary material for: Estimating direct and spill-over impacts of political elections on COVID-19 transmission using synthetic control methods
Source: PLoS Comput Biol. 2021 May 27;17(5):e1008959. doi: 10.1371/journal.pcbi.1008959 (PMC8158864; doi:10.1371/journal.pcbi.1008959)
Supplement: S1 Text — The figures were created using the base layer from The Humanitarian Data Exchange. Table A. Malaysia policy summary. Fig A. 95% Confidence intervals for treatment/spill-over effects at the district level. Fig B. Matching variables used for generation of synthetic controls (Healthcare capacity). Fig C. Matching variables used for generation of synthetic controls (Demographic/Geographic). Fig D. Sensitivity analysis on matching characteristics: magnitude of treatment/spill-over effects. Fig E. Sensitivity analysis on spill-over matrix, number of significant regions. Fig F. Sensitivity analysis on spill-over matrix, number of regions with positive treatment effects. Fig G. Sensitivity analysis on spill-over matrix, cumulative treatment effect. Fig H. Placebo test for treatment and spill-over effects prior to Sabah election. (DOCX) [file pcbi.1008959.s001.docx]

**Table A: Malaysia policy summary**

| **Date (2020)** | **Control measure** | **Affected states** |  |
| --- | --- | --- | --- |
| **January** | | | |
| 25 January | First three cases reported | Nationwide |  |
| 30 January | MOH published guidelines and establishes designated COVID-19 management hospitals nationwide | Nationwide |  |
| **February** | | |  |
| 27 February – 1 March | A Tablighi Jamaat conference was held in a mosque in Sri Petaling, with over 16,000 attendees from Malaysia and overseas | Selangor |  |
| 21 February – 1 March | The “Sheraton Move”: the Malaysian political crisis. The then coalition government collapses and a new coalition government is formed. A new Prime Minister is appointed. This seeded the 2020 Sabah state election. | Nationwide |  |
| **March** | | | |
| 11 March | Cases resulting from Tabligh cluster lead to second wave of infections in the country | Nationwide |  |
| 18 March | Movement Control Order (MCO)^1^ enacted | Nationwide |  |
| 27 March – 20 April | Enhanced MCO (EMCO)^2^ enacted in areas with new clusters | Johor, Selangor |  |
| **April** | | | |
| 10 April | MCO extended until 28 April | Nationwide |  |
| **May** | | | |
| 4 May – 8 May | Conditional MCO (CMCO)^3^ enacted nationwide with some exceptions; most economic sectors reopen with social distancing measures, continued ban on mass gatherings and interstate travel | Nationwide; except Kedah, Kelantan, Negeri Sembilan, Pahang, Penang, Perak, Sabah, Sarawak, Selangor |  |
| 14 May | Some areas under semi-enhanced MCO (SEMCO) | Kuala Lumpur |  |
| **June** | | | |
| 9 June | CMCO ends; announcement that country will enter Recovery MCO (RMCO) between 10 June and 31 August | Nationwide |  |
| 10 June – 31 December | Recovery MCO (RMCO)^4^ enacted nationwide; resumption of interstate travel and some religious gatherings | Nationwide |  |
| 24 June | Schools across Malaysia begin reopening, priority given to students taking secondary/international leaving exams | Nationwide |  |
| **July** | | | |
| 1 July | Government and private pre-schools, kindergartens, nurseries and day care centres resume operations | Nationwide |  |
| 27 July | Inter-zone travel is limited | Sarawak |  |
| 29 July | Commencement of political struggle between the former-former Chief Minister Musa Aman and then-Chief Minister Shafie Apdal; Musa Aman claims to have the majority in state assembly | Sabah |  |
| 30 July | Then-Chief Minister Shafie Apdal obtains the Sabah governor’s permission to dissolve the state assembly, paving way for fresh polls | Sabah |  |
| **August** | | | |
| 1 August | Mandatory face mask-wearing in public places introduced following emergence of new clusters | Nationwide |  |
| 3 August – 13 October | EMCO enforced in areas with new clusters | Kedah, Sabah, Selangor |  |
| 9 August | Announcement of 26 September as Sabah state election date | Sabah |  |
| **September** | | |  |
| 12 September | Nomination day – strict control measures implemented to prevent crowding | Sabah |  |
| 12 September – 25 September | Election campaigning period; political rallies take place across the state | Sabah |  |
| 14 September | Emergence and spread of community cases detected | Sabah |  |
| 26 September | Election day* | Sabah |  |
| **October** | | | |
| 5 October – 14 October | CMCO enforced in areas with new clusters | Sabah, Selangor |  |
| 8 October | Health Director-General announces start of the third wave of infections in the country | Nationwide |  |
| 14 October | Inter-district movement prohibited till 27 October | Selangor |  |
| 22 October | Employees in private and public sectors in the management/supervisory levels instructed to work from home | Selangor |  |
| **November** | | | |
| 9 November | CMCO reinstated nationwide with some exceptions | Nationwide; except Kelantan, Pahang, Perlis |  |
| 9 November | EMCO extended over several areas with new clusters | Negeri Sembilan, Sabah, Sarawak, Selangor |  |
| ^1^Movement Control Order   - Prohibition of movement and mass assembly nationwide, including all religious sports, social and cultural activities - All religious activities in mosques suspended, including Friday prayers - Malaysian citizens barred from leaving the country; restrictions placed on the entry of non-Malaysian citizens into the country - All sectors and businesses closed except for infrastructure services and stores selling daily necessities (e.g. supermarkets, grocery stores) - All educational institutions and vocational training centres closed | | |  |
| ^2^Enhanced Movement Control Order (put in place for 14 days if a large cluster was detected within the area to allow thorough COVID-19 tests and to curb the spread of the virus)   - All residents and visitors within the area forbidden from leaving their homes - Non-residents and visitors outside the area cannot enter the area - All businesses shut down - Adequate food supply provided by the authority during the 14-day order to all residents - Medical base will be established within the area - All roads into the area are blocked | | |  |
| ^3^Conditional Movement Control Order (relaxation of Movement Control Order regulations)   - Most economic sectors and activities allowed to open and operate with control measures in place, such as social distancing and recording the names, contact information and dates of visit of customers for contact tracing purposes - Outdoor sports activities that do not involve body contact, in small groups without audience and involving >10 persons are allowed with social distancing - Social, community, cultural or religious activities which involve large gatherings are still not allowed - Interstate travel only allowed for work purposes or to return home from another state | | |  |
| ^4^Recovery Movement Control Order   - Family entertainment outlets including arcades, cinemas and karaoke venues allowed to operate; other recreational activities such as clubbing and concerts are not allowed - Wedding ceremonies are allowed with fewer than 200 people in attendance at a time - Interstate travel allowed to resume - Employees are allowed to return to offices; students are allowed back in schools | | |  |

**Fig A: 95% Confidence intervals for treatment/spill-over effects at the district level**

Supplementary Figure A: I) Lower bound for the treatment/spill-over effect of the Sabah elections on COVID-19 case counts after the elections occurred, before reinstatement of nationwide conditional movement control order II) Upper bound for the treatment/spill-over effect of the Sabah elections on COVID-19 case counts after the elections occurred, before reinstatement of nationwide conditional movement control order. The figure was created using the base layer from The Humanitarian Data Exchange [1].

**Fig B: Matching variables used for generation of synthetic controls (Healthcare capacity)**

Supplementary Figure B: I) Total number of assistant medical officers in 2018 across districts II) Total number of medical rehabilitation officers in 2018 across districts III) Total number of medical officers/specialists in 2018 across districts IV) Total number of hospitals both public/private) in 2016 IV) Total number of specialised medical institutes in 2016. The figure was created using the base layer from The Humanitarian Data Exchange [1].

**Fig C: Matching variables used for generation of synthetic controls (Demographic/Geographic)**

Supplementary Figure C: I) Proportion Bumiputera across states II) Proportion Bumiputera across states III) Range in accessibility (minutes) across districts IV) Mean accessibility (minutes) across districts V) Total number of active land vehicles in 2018 across states IV) Total number of non-active land vehicles in 2018 across states . The figure was created using the base layer from The Humanitarian Data Exchange [1].

**Fig D: Sensitivity analysis on matching characteristics: magnitude of treatment/spill-over effects**

Supplementary Figure D: I) Number of spillover/treatment significant averaged over 10000 matching simulation runs II) Cumulative treatment direction averaged over 10000 matching simulation runs III) Cumulative treatment effect averaged over 10000 matching simulation runs. The figure was created using the base layer from The Humanitarian Data Exchange [1].

**Fig E: Sensitivity analysis on spill-over matrix, number of significant regions**

Supplementary Figure E: I) Spill-over Matrix 1 number of significant timepoints (Geometric flight distance between each region’s primary airport to Sabah’s primary airport) cumulative treatment effect II) Spill-over Matrix 2 number of significant timepoints (Geometric normalized number of flight passengers between each region’s primary airport to Sabah’s primary airport in October 2012) III) Spill-over Matrix 3 number of significant timepoints (Geometric normalized number of flight passengers between each region’s primary airport to Sabah’s primary airport from 2010 to 2017) . The figure was created using the base layer from The Humanitarian Data Exchange [1].

**Fig F: Sensitivity analysis on spill-over matrix, number of regions with positive treatment effects**

Supplementary Figure F: I) Spill-over Matrix 1 positive treatment regions (Geometric flight distance between each region’s primary airport to Sabah’s primary airport) cumulative treatment effect II) Spill-over Matrix 2 positive treatment regions (Geometric normalized number of flight passengers between each region’s primary airport to Sabah’s primary airport in October 2012) III) Spill-over Matrix 3 positive treatment regions (Geometric normalized number of flight passengers between each region’s primary airport to Sabah’s primary airport from 2010 to 2017). The figure was created using the base layer from The Humanitarian Data Exchange a[1].

**Fig G: Sensitivity analysis on spill-over matrix, cumulative treatment effect**

Supplementary Figure G: I) Spill-over Matrix 1 cumulative treatment effect (Geometric flight distance between each region’s primary airport to Sabah’s primary airport) cumulative treatment effect II) Spill-over Matrix 2 cumulative treatment effect (Geometric normalized number of flight passengers between each region’s primary airport to Sabah’s primary airport in October 2012) III) Spill-over Matrix 3 cumulative treatment effect (Geometric normalized number of flight passengers between each region’s primary airport to Sabah’s primary airport from 2010 to 2017) . The figure was created using the base layer from The Humanitarian Data Exchange [1].

**Fig H: Placebo test for treatment and spill-over effects prior to Sabah election**

Supplementary Figure H: I) Number of time points which reject the hypothesis of treatment/spill-over effects at the 95% level 8 weeks before the actual Sabah state election II) Number of time points which reject the hypothesis of treatment/spill-over effects at the 95% level 7 weeks before the actual Sabah state election III) Number of time points which reject the hypothesis of treatment/spill-over effects at the 95% level 6 weeks before the actual Sabah state election III) Number of time points which reject the hypothesis of treatment/spill-over effects at the 95% level 5 weeks before the actual Sabah state election III) Number of time points which reject the hypothesis of treatment/spill-over effects at the 95% level 4 weeks before the actual Sabah state election. The figure was created using the base layer from The Humanitarian Data Exchange [1].

**Reference**

1. Humanitarian Date Exchange. Malaysia - Subnational Administrative Boundaries. United Nations Office for the Coordination of Humanitarian Affairs; 2020. Available: https://data.humdata.org/dataset/malaysia-administrative-level-0-2-boundaries?
